# The role of habitat configuration in shaping animal population processes: a framework to generate quantitative predictions

**DOI:** 10.1007/s00442-021-04967-y

**Published:** 2021-06-22

**Authors:** Peng He, Pierre-Olivier Montiglio, Marius Somveille, Mauricio Cantor, Damien R. Farine

**Affiliations:** 1 Department of Collective Behavior, Max Planck Institute of Animal Behavior, Konstanz, Germany; 2 Centre for the Advanced Study of Collective Behaviour, University of Konstanz, Konstanz, Germany; 3 Department of Biology, University of Konstanz, Konstanz, Germany; 4 Department of Evolutionary Biology and Environmental Science, University of Zurich, Zurich, Switzerland; 5 Department of Biological Sciences, University of Quebec at Montreal, Montreal, QC Canada; 6 Birdlife International, The David Attenborough Building, Cambridge, UK; 7 Department of Biology, Colorado State University, Fort Collins, CO 80523 USA; 8 Department for the Ecology of Animal Societies, Max Planck Institute of Animal Behavior, Konstanz, Germany; 9 Departamento de Ecologia e Zoologia, Universidade Federal de Santa Catarina, Florianópolis, Brazil

**Keywords:** Habitat configuration, Habitat networks, Landscape connectivity, Movement networks, Social networks

## Abstract

By shaping where individuals move, habitat configuration can fundamentally structure animal populations. Yet, we currently lack a framework for generating quantitative predictions about the role of habitat configuration in modulating population outcomes. To address this gap, we propose a modelling framework inspired by studies using networks to characterize habitat connectivity. We first define animal habitat networks, explain how they can integrate information about the different configurational features of animal habitats, and highlight the need for a bottom–up generative model that can depict realistic variations in habitat potential connectivity. Second, we describe a model for simulating animal habitat networks (available in the R package *AnimalHabitatNetwork*), and demonstrate its ability to generate alternative habitat configurations based on empirical data, which forms the basis for exploring the consequences of alternative habitat structures. Finally, we lay out three key research questions and demonstrate how our framework can address them. By simulating the spread of a pathogen within a population, we show how transmission properties can be impacted by both local potential connectivity and landscape-level characteristics of habitats. Our study highlights the importance of considering the underlying habitat configuration in studies linking social structure with population-level outcomes.

**Supplementary Information:**

The online version contains supplementary material available at 10.1007/s00442-021-04967-y.

## Introduction

Animals rarely move unrestrictedly, as the physical habitat environments they depend on are often heterogeneous and uneven (Fahrig 2007; Kovalenko et al. 2012; Lovett et al. 2005). The physical configuration of habitats, such as the spatial arrangement of habitat components and their physical attributes (e.g. heterogeneity, size, and quality), can fundamentally determine the patterns of habitat potential connectivity (i.e. where animals of a species can go), which eventually determine how populations of given species are functionally connected (e.g. socially or genetically). Thus, habitat configuration can have broad implications for population and community dynamics across spatial and temporal scales, including ecological interactions (Jordano 2016; Plitzko and Drossel 2015; Ryser et al. 2019), community structure (Altermatt and Holyoak 2012; Henriques-Silva et al. 2013; Wilson et al. 2016), and speciation (Naka and Brumfield 2018). Habitat configuration can also determine the rates of social interactions among conspecifics, thus shaping the social structure of populations (Emlen and Oring 1977; Farine and Sheldon 2019; Gosling 1991; He et al. 2019; Leu et al. 2016). Ultimately, the physical configuration of habitats shapes the distributions of genes (Armansin et al. 2020; Beninde et al. 2016; Phillipsen and Lytle 2013), pathogens (Altizer et al. 2003; Loehle 1995; Silk et al. 2019), and information (Aplin et al. 2015; Laiolo and Tella 2005, 2006) in populations. Understanding the effects of habitat physical configuration on animal population and community dynamics is particularly important in a rapidly changing world, where natural populations increasingly face anthropogenic habitat changes.

How individual animals are socially structured has many consequences for populations (Allen et al. 2017; Aplin et al. 2012; Keeling 1999; Montiglio et al. 2018). The best example for this perhaps comes from studies on pathogen transmission (Cantor et al. 2020; Prado et al. 2009; Sah et al. 2018; Silk et al. 2019) describing how patterns of social or physical connections among individuals at local and global scales can impact the speed of transmission and the magnitude of disease outbreaks. Specifically, more clustered connections—where the number of shared social connections between individuals, or triads $A\leftrightarrow B$, $B\leftrightarrow C$, and $A\leftrightarrow C$, are more represented in the population—can increase the local spread (among immediate contacts) but decrease the speed and global reach of pathogen transmission (Keeling 2005; Read and Keeling 2003; Sah et al. 2018). However, to unravel the role of social structure in shaping ecological and evolutionary dynamics, we need to also understand the mechanisms that shape animal social structure. Alongside social decisions, features of the physical habitat environments can play a major role in shaping where animals move, who they (re-)encounter, and how often they interact with one-another (He et al. 2019). For example, a study in sleepy lizards (*Tiliqua rugosa*) found that habitats with more barriers increased the rates of encounters among individuals, increasing the density and clustering of the social networks (Leu et al. 2016), which may have implications for the spread of infectious pathogens (Tildesley et al. 2010; White et al. 2018). Early socioecological models have linked the spatiotemporal distribution of resources and risks to social behaviour (van Schaik 1989; Wilson 1975), while more recent models have focussed the behavioural mechanisms underlying social structure (Cantor and Farine 2018; Farine 2019; Ilany and Akçay 2016; Kappeler 2017; Spiegel et al. 2016). However, we also require quantitative tools that explicitly link configurational properties of habitats to social structures to enable us to generate testable hypotheses on the role of the physical habitat environments on socially mediated population outcomes.

The features of animal habitats are typically multi-faceted—they can be described by the heterogeneity, sizes, abundance and spatial arrangements of habitat components (Tokeshi and Arakaki 2012). For a given animal species, these features determine habitat potential connectivity, indicating where individuals can move, thereby, the behaviours that they express and the subsequent consequences for populations (Gilarranz et al. 2017). For example, Doherty et al. (2019) found that the shape of habitats, specifically whether habitats were wider (i.e. forming a rectangle) or thinner (forming a narrow strip), structured the movements of radio-tracked agamid lizards (*Pogona barbata*); specifically, activity area and daily movement rates were lower among individuals inhabiting thinner habitats. The actual movements of animals are then the outcomes of a range of drivers (Nathan et al. 2008), including habitat potential connectivity and individuals’ actual behavioural decisions (e.g. where to move for resources and/or mates), which over time determine how populations of a species are functionally connected (Calabrese and Fagan 2004; Tischendorf and Fahring 2000). Among these drivers, the spatial components inherent to many emergent ecological patterns have received increasing attention in ecology (Fletcher et al. 2013; Gilarranz et al. 2017).

Spatial networks (Barthélemy 2011) have been used to characterize metapopulation spatial structures and the spatial configuration of habitats (Dale and Fortin 2010; Fall et al. 2007; Urban and Keitt 2001; Urban et al. 2009), and the spatial patterns of connectivity of animal habitats (Alther and Altermatt 2018; Bodin and Norberg 2007; Fall et al. 2007; Galpern et al. 2011; Lookingbill et al. 2010; Marini et al. 2019; Minor and Urban 2008; Poli et al. 2020; Urban and Keitt 2001; Urban et al. 2009). For example, Robertson et al. (2018) used long-term mark–resight data to construct networks that characterize the functional connectivity among habitat patches of snail kite (*Rostrhamus sociabilis plumbeus*) to evaluate the relative roles of among-patch movement and reproduction in modulating the effective connectivity of the species’ distribution range. In such networks, nodes often represent habitat or resource patches (e.g. nesting sites, Galpern et al. 2011; Urban et al. 2009), that is, areas crucial for survival and reproduction (Fahrig and Merriam 1985) as opposed to the landscape matrix (Ziolkowska et al. 2014). How these connections are defined determines what these networks are depicting. Typically, the connections are inferred from movements of individuals, gene flow, the species’ biological attributes, or from the characteristics of the environment itself (see Calabrese and Fagan 2004 for the definitions of connectivity metrics; but see review on the use of these connectivity metrics in Galpern et al. 2011).

**Fig. 1:**
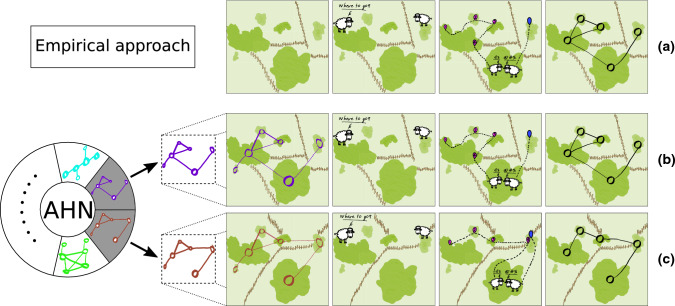
Two distinct approaches for understanding the role of habitat configuration in shaping animal population (or community) structures. **a** In most studies, animals are observed living and moving (e.g. via GPS tracking) within given time windows in specific habitats, from which characteristics of the connectivity of the focal habitat area are inferred or modelled (e.g. by resistance surface modelling, network-based landscape connectivity modelling, or circuit theory). By contrast, (**b**) with a bottom–up approach, we can simulate networks to depict the physical configurations of specific habitats, and then model individual movements (or more complex behaviours) in these habitats, from which we can gain sights on how observed structures (e.g. patterns of movements and social interactions) emerged. With this approach, we can also (**c**) simulate habitat networks controlling for key parameters (e.g. network connectivity), thus producing alternative scenarios that can control (or not) for features that are hypothesized to play a major role in shaping biological processes in populations. Here, we illustrate two simulated networks, one of which (**b**) can exactly depict the configuration of the given habitat for the focal species (**a**), while the other depicts a habitat that maintains some characteristics (e.g. the same distributions and sizes of habitat patches, represented by nodes) as the given habitat (**a**), but provides alternative patterns of potential connectivity (by randomizing the spatial distribution of movement barriers that determine which patches are connected)

Spatial networks constructed following the approaches outlined above have been instrumental in studies of animal movements (Gilarranz et al. 2017; Robertson et al. 2018) and community structure (Altermatt and Holyoak 2012). However, they typically do not allow us to make broader, or more general, predictions on the linkages between spatial network structures and processes in animal populations and communities. This is because the structures of empirically constructed spatial networks are usually derived from, and thus inherently limited in scope by, the specific habitats and species under study (Baguette et al. 2013; Baranyi et al. 2011; e.g. Fig. 1a). Thus, they limit our understanding on how various configurational features of habitats (e.g. landscape linearity and spatial scale) might consistently shape population or community outcomes. For example, networks explicitly built to characterize the potential connectivity of the Yangtze and the Rhine rivers for freshwater organisms can be used to explore the relationships between these specific network structures and processes in populations or communities. Yet, the specificity of such networks, as originated from the specific spatial configurational features (e.g. landscape geometry, elevation) of those habitat systems for targeted species, may limit our ability to explore a broader spectrum of plausible habitat networks, including those that might not yet exist but could emerge from present habitat networks (e.g. through rerouting of river flow or the construction of a dam). Moreover, empirical networks provide limited ability to explore how specific dimensions of network properties (e.g. patterns of connectivity) affect populations while holding other properties constant (e.g. network size, spatial scales at which these networks are defined; e.g. Fig. 1b, c). For example, empirical networks have suggested that highly modular social structures (i.e. multilevel social structures) play an important role in promoting cultural evolution (Migliano et al. 2020); yet, it has been subsequently shown that such effects of modularity contribute relatively little in promoting cultural evolution by simulated networks, which allow testing the effects of network connectivity itself while controlling other network properties such as the number of links (Cantor et al. 2021). Such limitations may make it hard to generalize insights and predictions on the relationship between the physical habitat environment and biological processes.

One way to overcome the limitations of empirical networks is to simulate networks using generative models (Granovetter 1973; Watts and Strogatz 1998). While many generative models exist (e.g. Barabási et al. 2001; Erdős and Rényi 1960), none considers the inherent spatial dependency of animal habitat networks, and thus generate networks might not capture their fundamental properties (but see Carraro et al. 2020 for a recent solution for riverine habitats). One generative model of random networks that can incorporate spatial components is the random geometric network model (Dall and Christensen 2002; Penrose 2003). In geometric networks, nodes are anchored in space and are connected whenever their Euclidean distance is below a given threshold. By producing distance-based spatial networks (Barthélemy 2011), the random geometric network model provides a starting point for generating habitat networks. However, random geometric networks remain limited by a fixed spatial extent (i.e. a consistent square landscape) and a fixed threshold for determining the presences of links. In nature, the geometry of habitats and landscapes for a species, and the topological properties of their potential connectivity, can vary widely. For example, the distance between two patches does not exclusively dictate their potential connectivity—barriers such as waterscapes can restrict movement of a terrestrial animal between two patches in close proximity, while patches that are far apart can be connected by movements (as demonstrated by empirical evidence that primates can use roads to efficiently move between distant areas of their home ranges, Green et al. 2020; Strandburg-Peshkin et al. 2017).

Here, we address the need for a generative model of animal habitat networks by extending the random geometric network model to generate more plausible spatial networks. We first define animal habitat networks and outline the key configurational features of animal habitats that can be captured by such networks. Next, we describe a modelling framework—available in the accompanying R package *AnimalHabitatNetwork,* for simulating animal habitat networks explicitly tailored to depict the diverse physical configurations of animal habitats. We show that our network simulation algorithm can be tuned to capture the patterns of potential connectivity of real habitats efficiently, thereby providing the basis for explorations of alternative scenarios. Doing so is important, as making predictions requires producing realistic alternative scenarios. We propose three key research questions related to our modelling framework. Finally, we illustrate how our framework can be used to simulate animal habitat networks with varying patterns of connectivity to investigate the implications of habitat configuration for populations by embedding a Susceptible-Infected-Recovered (SIR) epidemic model in our modelling framework. Taken together, our findings provide new insights on the linkages between habitat configuration and population-level outcomes and highlight how the application of an explicit and quantitative framework to simulate habitat networks can help us gain a better mechanistic understanding of the role of habitat configuration in shaping the dynamics of ecological, evolutionary processes and their conservation implications.

## A multi-dimensional framework for modelling animal habitat configuration

### Defining animal habitat networks

**Fig. 2:**
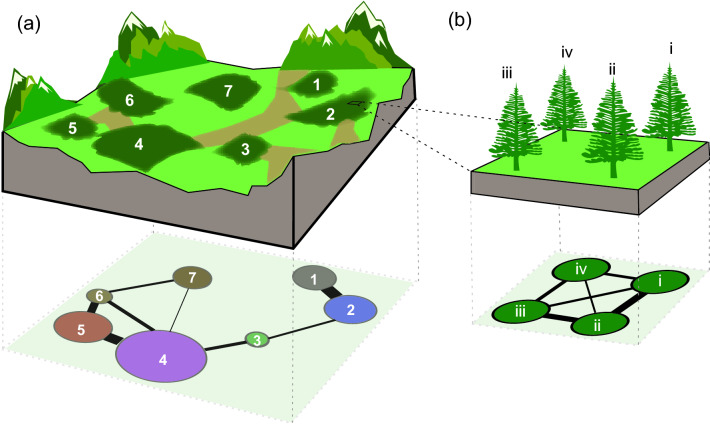
Networks explicitly characterizing the physical configurations of animal habitats. We illustrate how five dimensions for assessing habitat configuration proposed by Tokeshi and Arakaki (2012) can be integrated and applied to construct animal habitat networks. These dimensions are (1) *spatial scale* (spatial resolution and extent), (2) *composition diversity* (heterogeneity), (3) *size* (area), (4) *abundance or density* (number of discrete habitat units per area), and (5) *spatial arrangement* (distribution) of habitat components. (**a**) A hypothetical landscape composed by forest fragments (numbered components) within a heterogeneous matrix with potential movement corridors (light green, which account for the presences of links between nodes) and physical barriers (light brown, which account for the absence of links between nodes). The physical features and spatial organization of the habitat components can be represented by a connected network at a large spatial scale, with a high composition diversity (fragments of different tree species), different habitat sizes (small and large fragments), high abundance (7 fragments), and heterogeneous spatial arrangement (fragments unequally distributed and connected by movement corridors across the landscape). (**b**) The physical features and spatial arrangement of habitat components can be characterized at different spatial scales. Here, part of the forest (fragment 2) can be represented by a connected network at a finer spatial scale (e.g. trees as habitat components), with a low composition diversity (the same tree species), small habitat size (single trees), low abundance of components (4 trees), and uniform spatial arrangement. In the two habitat networks, the compositional diversity (or quality) and size (or carrying capacity) of habitat components are characterized by node attributes (colours and sizes), the abundance by the number of nodes in the networks, and the spatial arrangement by the patterns of connectivity and the distribution of link weights (both as a function of the Euclidean distances between habitat components)

Animal habitats are defined by taking both the species-level properties (such as locomotion and space use characteristics of a focal species) and the environmental features into account. Here, we highlight the five fundamental dimensions proposed by Tokeshi and Arakaki (2012) for characterizing habitat physical configuration as a means of defining components in animal habitat networks. These dimensions are (1) *spatial scale* (spatial resolution and extent) at which the landscape and its elements are defined, (2) *composition diversity* (heterogeneity), (3) *size* (area), (4) *abundance or density* (number of discrete habitat patches/units per area), and (5) *spatial arrangement* (distribution) of habitat components. With these dimensions and following the definitions of connectivity metrics (i.e. structural, potential, and realized connectivity; Calabrese and Fagan 2004; Taylor et al. 1993; Tischendorf and Fahring 2000; Urban and Keitt 2001), we define animal habitat networks (Fig. 2) as network-based explicit depictions of (1) *the spatial organization* and (2) *the physical attributes* (e.g. *heterogeneity* and *area*) of given *numbers* of habitat components at given *spatial scales*, and (3) *the potential connectivity* indicating where animals of a given species can move.

In habitat networks, nodes represent spatially explicit habitat components (or patches) which can be characterized by attributes (e.g. size, quality, and physical composition). The presence of a link between nodes indicates that animals of a given species can move between patches. In weighted animal habitat networks, link weights can characterize variation in the propensity for individuals to move between patches. Variation can arise from a range of factors, such as the spatial proximity between habitat components or the permeability of the landscape matrix between habitat components. Link weights can also be defined by empirical data (such as the actual rate of movement or gene flow between patches previously observed, which are typically treated as measurements of the extent to which landscapes facilitate/impede movements of individuals of a species, Taylor et al. 1993). Link presences and weights can also be related to properties of habitat patches (i.e. node attributes), such as the extent to which they are similar in their attributes for a species (e.g. types of resources they provide for a species).

The definition of the presences and weights of links in habitat networks highlights a clear distinction between network structures defined as a priori potential connectivity versus those determined by post hoc observational data. The former provides a fundamental template indicating where animals of a species can move, as the outcome of how the species’ intrinsic biological attributes (e.g. locomotion, Hirt et al. 2018) interact with the configurational features of the physical habitat environment. By contrast, the latter reflects realized animal movements (e.g. animal movement networks; see properties of movement networks in Bastille-Rousseau et al. 2018; Jacoby and Freeman 2016) that are typically driven by habitat potential connectivity together with a range of factors. These include the social environment that contribute independently to individuals’ movement decisions (Armansin et al. 2020; Strandburg-Peshkin et al. 2015, 2017), but also methodological factors (e.g. measurement accuracy, effort, decisions about where to collect data). Defining animal habitat networks from the perspective of potential connectivity highlights the fundamental bottom–up role of habitat physical configuration together with focal species’ biology in structuring animal movements and the subsequent processes in populations or communities.

### The AHN model

**Fig. 3:**
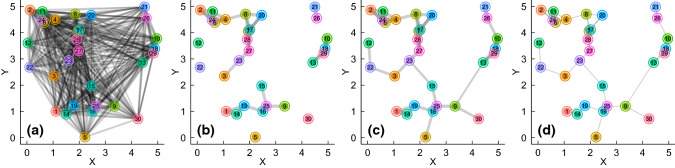
The workflow of the AHN model for generating animal habitat networks. First, (**a**) the algorithm constructs a fully connected and weighted habitat network. Here, numbered nodes represent 30 habitat components colour-coded by their attributes (such as their sizes, quality or compositions, with continuous or discrete colour palette) and connected by links whose thicknesses indicate the strength of the spatial relationship between the two habitat components, and is determined by the spatial positions of the nodes. The network is defined in a conceived 2-dimensional landscape in which the $x$ and $y$ axes indicate the spatial extents of the landscape (here the aspect ratio is 1, i.e. $A={L}^{2}$, but the model allows any $x$ and $y$ extents for capturing the diverse landscape geometry), therefore, it inherits spatial properties of the landscape. Next, (**b**) the algorithm removes the link between node $i$ and $j$ ($i\ne j$) from the network with probability ${P(D}_{ij})$; in this example, it results in a disconnected habitat network. Then, (**c**) the (disconnected) network components can be rewired with minimal number of links if a connected network is wanted. Finally, (**d**) the habitat network can be transformed to unweighted, if so desired (e.g. when we are interested only in the patterns of potential connections while their attributes are irrelevant to our hypotheses)

We propose a general and spatially explicit modelling framework for plausible spatial networks (hereafter the ‘AHN’ model) that can depict the diverse configurational features of animal habitats. Although we focus on animals, our framework can equally be applied to characterize the configurational features of habitats for any moving organisms, such as pathogens in moving hosts and dispersing seeds. The model contains eight parameters (Table 1) explicitly encoding five fundamental dimensions characterizing animal habitat physical configuration (Tokeshi and Arakaki 2012) and species-level movement characteristics. We define the model within a 2-dimensional Euclidean space, by conceiving a planar rectangular landscape with an area $A$ and a side length $L$. The model can accept any given spatial layouts of habitat components. For example, the coordinates can be determined by the spatial locations of empirically observed natural habitats, approaches developed for simulating point patterns in spatial ecology (Baddeley et al. 2016; see also Baddeley and Turner 2005 for the R package ‘spatstat’), or any spatial distributions relevant to a hypothesis of interest (e.g. the layout simulated using the Gauss-Poisson point process; see  also the example in Code Availability). By default, the spatial coordinates $\boldsymbol{x}$ and $\boldsymbol{y}$ of the $N$ habitat components are randomly drawn from the intervals $[0,L]$ and $[0,A/L]$ (e.g. Fig. 3a), respectively. In this way, the AHN can depict landscapes with variable sizes, spatial extents, and aspect ratios, which can be based on the geometric properties of empirical animal habitats. Thus, the model explicitly captures the number (or density) of habitat components at the given spatial scale. Finally, in the model, the compositional diversity (i.e. heterogeneity) and size (or other physical properties) of habitat components can be encapsulated as node attributes, in vectors $\boldsymbol{U}$ and $\boldsymbol{V}$ respectively, the values of which can be quantitative or qualitative, and can be provided specifically or drawn at random from a given distribution, depending on the hypothesis of interest. By being explicit in spatial scale and node attributes (e.g. heterogeneity), the model can relate spatial scaling (Fletcher et al. 2013) to the functionality of the physical habitat environment for organisms of focal species (Fahrig et al. 2011).

**Table 1:** Parameters of the AHN model for depicting habitat physical configuration

Parameter	Description
$A>0$	Area of the conceived landscape
$L>0$	The length of one side of the conceived landscape
$N>0$	Number of habitat components (integer)
$\eta \ge 1$	Scaler for the weights of rewiring links
$\lambda >0$	Determining the steepness of link filtering-out function ${P(D}_{ij})$
$\varvec{\mu}$	Determining the concave-to-convex transition point of ${P(D}_{ij})$
$\varvec U$	Heterogeneity (or qualitative properties) of habitat components
$\varvec V$	Sizes (or quantitative properties) of habitat components

Once the layout of habitat components is defined, the AHN model can then generate links to characterize the patterns of potential connectivity among habitat components for a given species. Links can be weighted (e.g. Fig. 3c, with link weights characterizing the strength of connections) or unweighted (e.g. Fig. 3d, where the strength of connections is not of interest), and are non-directed (the model can easily be extended to have directed links, for example if there is a gradient—such as altitude—a physical features of habitat that might favour animal movements in one direction more than the other).

When generating links, the model starts by allocating a link weight between node $i$ and node $j$ ($i\ne j$). By default, the model uses the power function $W\left({D}_{ij}\right){=D}_{ij}^{-1}$, where ${D}_{ij}$ is the Euclidean distance, a primary metric characterizing habitat potential connectivity (Poli et al. 2020), between habitat components that node $i$ and node $j$ are referring to (Fig. 3a). However, alternative approaches can be used to define link weights, such as relating them to the similarities or differences in the properties of patches themselves (i.e. node attributes characterized by $\boldsymbol{U}$ and/or $\boldsymbol{V}$, such as their heterogeneity), using different metrics such as cost-distance (Fletcher et al. 2016), using dispersal kernels (Clobert et al. 2012; Hartfelder et al. 2020; Urban and Keitt 2001; see also examples in Code Availability), or using information on habitat choice of a species, such as evidence that animals can preferentially disperse to habitats with similar properties as their natal habitats (Davis 2008; Hoover et al. 2021)—which would mean that patches with greater similarity would have a stronger link weight.

With our approach, the network starts by being fully connected, with link weights indicating the strength of potential connectivity between pairs of habitat components. In nature, however, animals are often limited by their ability to move between habitat components that could otherwise be connected by movements, for example, due to physical barriers to their movements, and/or spatial distances that they are intrinsically not able to directly cover between habitat components. To capture this realism, we use the sigmoidal function $P\left({D}_{ij}\right)={[1+\mathrm{e}\mathrm{x}\mathrm{p}(-\lambda ({D}_{ij}-\mu ))]}^{-1}$ to determine a threshold probability for *filtering out* the link between node $i$ and $j$ from the initial complete network, where $\lambda$ determines the steepness of the thresholding curve which transits from concave to convex at the species-specific critical distance ${D}_{ij}=\mu$. We define $\lambda >0$ so $P\left({D}_{ij}\right)$ consistently increases over ${D}_{ij}$ (Fig. A1). This function enables us to generate a wide spectrum of curves to cover the diverse and evolving relationships between ${D}_{ij}$ and $P\left({D}_{ij}\right)$ by tuning $\lambda$ and $\mu$ (Figs. A1–A3). The dependence of the probability on ${D}_{ij}$ assumes that it is less likely that there exists a *direct* movement potential between two habitat patches when they are much further away.

The probabilistic nature of the filtering function captures the stochasticity that exists in the relationships between spatial proximity, landscape configurational features and potential connectivity. That is, on one hand, $\mu$ operates on the distances between patches to characterize the baseline stochasticity as the product of the interplay between species-specific characteristics of how individuals typically move (e.g. locomotion mode or capacity) and the given spatial proximity between habitat components, while, on the other hand, $\lambda$ is a coefficient which characterizes the stochasticity arising from how the intrinsic biological properties of a species interact with the given configurational features of the physical habitat environment between habitat components with the given spatial proximity, such as the presences/absences of physical barriers, and/or the amount of resistance to movements. With $\mu$ and/or $\lambda$ we can generate the patterns of absences/presences of links that are ecologically relevant to our hypotheses or questions (e.g. parameterization with empirical or observational data on focal species’ attributes, spatial proximity between habitat components, and/or the quality of landscape matrix for the focal species).

Our framework provides a starting point for incorporating more complex approaches for simulating spatial networks as habitat networks. It provides a simple and flexible approach to simulating networks that can capture the diverse patterns of potential connectivity of animal habitats as found in nature. For example, with a given set of spatially referenced nodes, ${P(D}_{ij})$ allows us to simulate variable patterns of network connectivity, such as node clustering, which can be used as meaningful depictions of the potential connectivity of alternative scenarios of empirically derived habitats or to generate scenarios for habitats based on observed (or hypothesized) locomotion modes of a species. ${P(D}_{ij})$ can also determine the presence of links in both probabilistic and deterministic ways, which not only makes the framework general, but also enriches our ability to encode the diverse physical features between habitat components, such as the variation in the quality of habitat matrix (in terms of their effects on animal movements), and species’ intrinsic attributes, such as the ability to exploit the physical landscapes. For instance, in the extreme case when $\mu \to -\infty$ and $\lambda \to +\infty$, the link removal function becomes deterministic (i.e. ${P(D}_{ij})\to 1$), and with the rewiring option in the model (described below), the model can then generate networks that approximate planar networks (McDiarmid et al. 2005), which have previously been used to model landscape functional connectivity (Chubaty et al. 2020).

In some cases, ${P(D}_{ij})$ fragments the network into (disconnected) network components (e.g. Fig. 3b). The smaller the $\mu$ gets and the larger the $\lambda$ gets, the more links on average will be filtered out, and the more likely it is for the resulting habitat network to be disconnected (Fig. A3). In such cases, a disconnected network would represent habitats containing isolated clusters of habitat components between which animals cannot physically move among them. From a modelling perspective, it is often preferable (at least initially) to consider one habitat as a connected network (i.e. a single network component) which denotes a complete habitat or a section of a larger fragmented habitat in which individuals can theoretically (but not necessarily) move from one patch to any other. This means that the whole of the focal population can be functionally connected as a biologically meaningful unit (e.g. gene flow is possible between any two patches). We, therefore, incorporate the option of using a step-wise approach to rewire network components for connected habitat networks. In the rewiring, the two spatially closest nodes from each of the two network components are wired each time until the network has no disconnected network components, and the algorithm uses the minimum number of rewiring links for doing this (Fig. 3c). While by default the weights of rewiring links (if simulated) are defined in the same way as for the links within connected clusters, the model provides the option to additionally mediate these weights (for example, if we expect lower movement potentials between clusters, given the distance between them). We implement this option with the function $G\left({D}_{\alpha i\beta j},\eta \right)={D}_{\alpha i\beta j}^{-\eta }$, where ${D}_{\alpha i\beta j}$ is the Euclidean distance between node $i$ from the network component $\alpha$ and node $j$ from the network component $\beta (\alpha \ne \beta )$, and the scaler $\eta \ge 1$ enables control over the weights of the rewiring links (if any; Fig. A4), and by default $\eta =1$ (i.e. all the link weights in a network are defined with the mathematical reciprocal of the Euclidean distance between nodes).

We provide the implemented algorithm for simulating habitat networks in the function *ahn_gen()* in the R package *AnimalHabitatNetwork* (version 0.1.0, He and Farine 2019; see Code Availability).

### Demonstrating the capability of the AHN model in simulating habitat potential connectivity

The pattern of connectivity is the key signature of a network (Albert et al. 1999). To test the capability of the AHN model in simulating habitat networks that are similar in terms of their structural properties (i.e. connectivity) to those observed from real habitats, we compared the topological properties of networks generated by the model using a given parameter space with those of empirical habitat networks characterizing habitat potential connectivity by Friesen et al. (2019). Here we consider three network metrics, the (average) clustering coefficient, modularity and diameter. Studies have discussed the relationships between these metrics of (social) networks and population outcomes, such as transmission of pathogens (Sah et al. 2018) and evolutionary dynamics (Marcoux and Lusseau 2013; Raghunandan and Subramanian 2012) in populations. In the context of animal habitat networks, these metrics could explain outcomes where individual movement play a fundamental role. The clustering coefficient (Fagiolo 2007) in animal habitat networks characterize the probability that two patches connected to a third patch are themselves connected, which can capture the extent to which individuals are locally constrained and contained by the physical habitat environments. Modularity (Newman 2006) in animal habitat networks characterizes the extent to which clusters of habitat patches tend to be more densely connected with each other within the cluster than with other clusters, which can capture the extent to which individuals are facilitated in local movements but impeded in movements at larger spatial scales by configurational features of the physical habitat environments (where higher modularity would represent more distinct subpopulations). Diameter (Albert et al. 1999; Jackson 2008) in an animal habitat network captures the length of the longest (yet the most efficient) potential movement path between two patches within a given habitat, which can capture the linearity of habitat potential connectivity.

**Fig. 4:**
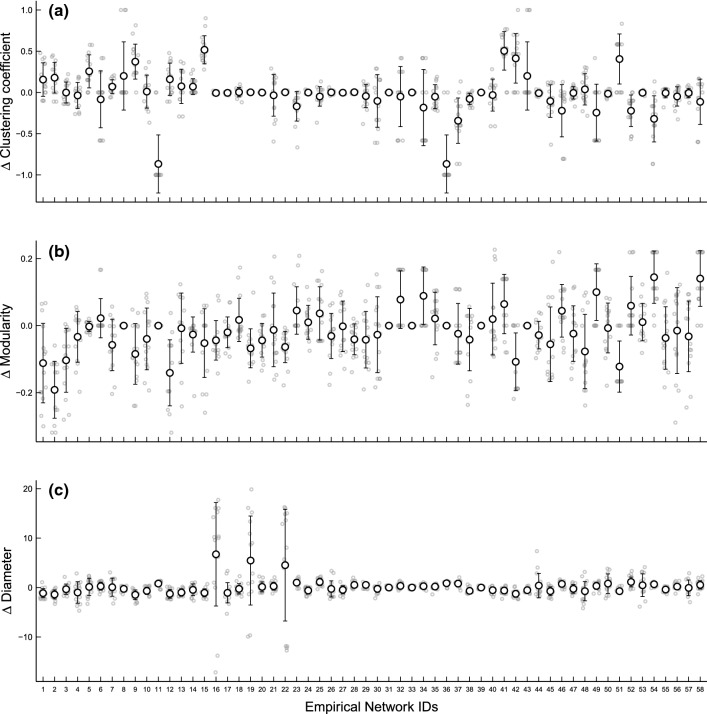
The AHN model can simulate spatially explicit networks to characterize habitat potential connectivity. Each grey circle denotes the difference in each of the three metrics (y-axes, **a**, **b**, **c**) between each of the 15 replicated random habitat networks generated by the AHN model with each set of best-fitting parameters identified from the given parameter space and the corresponding empirical network; black circles and bars characterize the means and the standard deviations

We extracted the largest network component from each of the 62 empirical habitat networks contained in the Friesen et al. (2019) dataset, and kept 58 of them for benchmarking (the two largest were omitted due to computation limit and the two smallest, each with two nodes, were excluded). Each of these extracted networks is connected, denoting a habitat or a part of a larger habitat where animals can physically move from a given habitat component to any other one (i.e. the habitat can be functionally connected—biological processes such as information or genes flows are possible among habitat components). Next, we simulated random habitat networks with the AHN model and identified those sets of parameters under which the corresponding output habitat networks best approximated the (average) clustering coefficient, modularity, and diameter of each of these empirical networks, respectively. We considered the parameter space $A=25$, $L\in \{\mathrm{5,10,15,20,25,30}\}$, $\mu \in \{\mathrm{0.1,2},\mathrm{5,7},10\}$, $\lambda \in \{\mathrm{0.001,0.1,0.15,0.35,0.4,0.75,1.25,5},30\}$ across all empirical benchmark networks, while keeping $N$ identical to the number of nodes of the corresponding empirical habitat network. This parameter space was determined by considering the effects of each parameter on the resulting network structures (Fig. A1–A7). In total, for each metric of each empirical network, we generated 270 (i.e. size of the parameter space) random habitat networks, and identified the set of parameters from the parameter space that generated the network that most closely approximated the metric of the given empirical network as the ‘best-fitting’ set of parameters for that network. We then simulated 15 habitat networks with each of these sets of parameters as replicates, and evaluated the extent to which each of these metrics of each replicate deviated from that of the corresponding empirical network (see Code Availability). The test of the model with these networks confirmed that our proposed algorithm can generate networks that capture the key structural properties of real habitats (Fig. 4), thereby forming the basis for subsequently exploring on how population outcomes (structures and/or processes) might change under alternative habitat scenarios (e.g. by controlling and/or parameterizing key parameters from empirical and/or observed landscapes, such as increasing or decreasing connectivity by tuning $\mu$ and/or $\lambda$). For example, if we wanted to test whether a species in a landscape with a given set of configurational features is more prone to infectious pathogens than another species, we can model transmission dynamics with epidemic models on habitat networks defined by species-specific $\lambda$ and/or $\mu$. All network computations were done in R (version 3.6.1, R Development Core Team 2019) with the *igraph* library (version 1.2.5, Csardi and Nepusz 2006).

## Key research questions related to the modelling framework

The AHN model can be used to address a range of topics and research questions. Here, identify three key research areas where our framework can be used to address outstanding questions.

### What are the consequences of habitat changes for population processes in social animals?

Animal habitats are changing under natural and anthropogenic drivers, typically characterized by changes in the spatial distribution of habitat components (e.g. food and shelter), changes in their potential connectivity (e.g. through fragmentation or reforestation), and/or changes in the physical attributes of the habitat components themselves (e.g. the amount of resources in each patch). These changes can then reshape the movements of animals, which can subsequently affect the patterns of biological or ecological interactions (e.g. inter-individual social structure, predator–prey interactions), or even impose evolutionary pressures on impacted species (Banks et al. 2011; Kokko and Sutherland 2001). The spatial distributions of food resources or habitat fragmentation can shape the spatial organization of individuals (Jacobson et al. 2015; Mourier et al. 2012), with consequences on the evolution of their social or mating systems (Banks et al. 2007; Emlen and Oring 1977; Tuomainen and Candolin 2011; van Schaik 1989). For example, Banks et al. (2011) empirically explored the relationship between the patterns of den-sharing interactions among hollow-dependent Australian mountain brushtail possums and the spatial variation in hollow tree availability, and found a behavioural switch from kin avoidance to kin preference in den sharing when hollow tree availability decreases, highlighting the important role of habitat change in driving individuals’ social behaviours as responses. In another example, Bain et al. (2014) examined the effects of habitat configuration on the frequency of extra-pair paternity (EPP) in cooperatively breeding superb fairy-wren (*Malurus cyaneus*) by linking spatial arrangements of their territories to the frequency of EPP, and found that the frequency of extra-group paternity (EGP) among groups in linear strips of vegetation was lower than those in more clustered territories in continuous habitats, highlighting the role of habitat spatial configuration in influencing the rates of EGP and the potential consequences of anthropogenic habitat change for mating systems.

Our network-based modelling framework can be used to depict multiple yet diverse configurational properties of animal habitats, thereby providing the starting point for explicitly modelling habitat change and predicting the population outcomes. Moreover, understanding the consequences of habitat fragmentation for populations is one of the central topics in conservation biology (Fischer and Lindenmayer 2007; Haddad et al. 2015), while the impacts on social processes of conservation efforts aimed at reducing habitat fragmentation are almost completely unexplored. With our framework, one can simulate network scenarios that realistically map the potential trajectories of habitat change (e.g. by parameter optimization and/or network manipulation), and generate predictions on the potential consequences for a population.

### How is habitat connectivity shaped by landscape and species properties?

The patterns of potential connectivity that form animals’ habitats are shaped by a range of properties. Some of these are biological, such as species attributes. For example, the movement capacity of an animal species can be driven by body mass and locomotion, influencing how they explore their physical habitat environments (Hirt et al. 2018). Many of the properties shaping potential connectivity are abiotic, such as the climatic conditions that determine the composition of habitat patches (e.g. the assemblage of plants, or coral, species in a patch) and geological features that determine the shape of the landscape (e.g. the long and narrow valleys created by a mountain range vs. an open plain). A key question is, therefore, whether certain types of landscapes consistently shape networks with different properties. For example, it is likely that riverine habitats, or habitats in valleys, will have a larger network diameter than habitats that are less restricted by the geometric features of landscapes. Studies have highlighted the importance of linking the configurational features of landscapes and species-level properties to population-level outcomes, and practical guidelines have been proposed for exploring such linkages (e.g. Frank and Wissel 1998). Using our framework and following a fundamental bottom–up approach, it will be possible to develop a mechanistic understanding of the relative roles of the multiple factors underlying population outcomes, such as species-level properties (e.g. body mass and locomotion characteristics, Hirt et al. 2018) and landscape properties (e.g. linearity) in shaping structural properties of habitat networks (e.g. network clustering).

### How different do we expect population social structures to be in different landscapes?

**Fig. 5:**
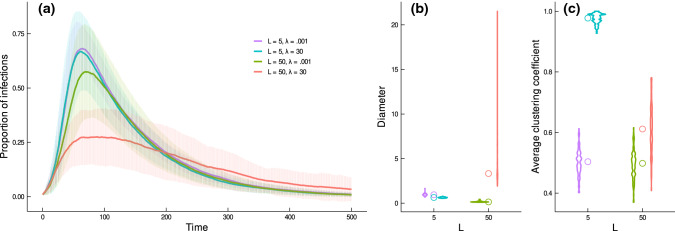
The application of the AHN model for understanding the role of habitat geometry and potential connectivity in mediating pathogen transmission dynamics in habitat-structured animal populations. **a** The transmission of pathogens in populations is dependent on both the landscape geometry (shape), depicted by $L$ where a larger value represents landscapes with a larger aspect ratio, and the extent to which the potential connections between patches to be determined by the habitat features between them, depicted by $\lambda$, where a lower value corresponds to a weaker deterministic effect of how the species’ movement characteristics interact with the environmental features on the potential connectivity between patches with given spatial proximity. Simulations show that the transmission dynamics, when individual mobility is at a medium level ($1-{p}_{s}=0.5$) under an infection rate of $\pi =0.05$ and a recovery rate of $\rho =0.01$, are impacted by habitat shape and potential connectivity. Specifically, habitats with a larger aspect ratio (a larger $L$ value) and with their potential connectivity determined with a stronger deterministic effect of the configurational features on the potential connectivity between patches with given spatial proximity (i.e. a larger $\lambda$ value) have the smallest disease outbreaks (each curve indicates the mean percentage of infected individuals in a population of 100 individuals moving on simulated habitat networks comprising 20 nodes, over 500 timesteps, with bars indicating the standard deviations from 100 replications). The interaction between landscape shape and the degree to which between-patch potential connectivity is determined by habitat configurational features affects the structural properties of habitat networks, such as diameter (**b**) and (average) clustering coefficient (**c**). Open circles (in **b** and **c**) indicate medians

Studies have revealed that animal population social structures often exhibit notable variations (Mori and Saito 2005; Nandini et al. 2017; Prehn et al. 2019; Whitehead and Kahn 1992). When habitats vary in their physical configurations, we would expect the social structures of populations in these habitats to exhibit variations (even for the same species), and this is indicated by empirical evidence. For example, Farine and Sheldon (2019) showed that the social network structure (at the network community level) of a woodland bird community observed in the Wytham Woods in the UK, remained consistent across four winters, despite the high turnover rate of individuals within the communities. This study suggests that the predictability of habitat configuration for the emergent social network structures. Our framework can be tuned to depict animal habitats with distinct configurational features, therefore, providing a theoretical tool to examine how much variations in population social structures observed from one habitat to another might be explained by habitat physical configurations (i.e. the extent to which animal habitat networks account for the variations in animal social networks). Figure 5 highlights how different aspects of habitat configuration (such as the aspect ratio and the tendency for distant patches to be connected versus not) can interact with each other to shape the resulting structural features of the habitat networks.

## Illustrating the application of the framework: how do habitat networks shape pathogen transmissions?

Our modelling framework can be used for understanding the link between animal habitat network structures and population outcomes. Here, we illustrate a possible application of it in the context of pathogen transmission in a population of 100 individuals (with no birth, death, emigration from and immigration to the landscape) moving among 20 habitat patches, where the patterns of potential connectivity are depicted by (connected) habitat networks simulated using the AHN model. We considered the simplest case where habitat patches are randomly positioned (with their $x$ and $y$ coordinates drawn from uniform distributions) in landscapes with the same area (i.e. $A=25$) but varying aspect ratios, with $L=5$ producing square-shaped landscapes (e.g. resembling forests on open plains) and $L=50$ producing landscapes with a large aspect ratio (e.g. resembling forests in a narrow valley). Within these landscapes, we considered two scenarios of habitat potential connectivity, defined by $\lambda =0.001$ and $\lambda =30$. These values depict two distinct outcomes of how the focal species’ intrinsic biological attributes interact with the environmental features between habitat patches, whereby $\lambda =0.001$ characterizes a weaker deterministic effect of such interactions on the potential connectivity between patches with given spatial proximity, and $\lambda =30$ represents a stronger effect. We maintained other parameters constant ($\mu =5$ and $\eta =1$).

We initiated simulations by randomly allocating individuals to the nodes of each habitat networks and modelled individual movements and transmission dynamics for 500 timesteps. When modelling individual movements, we defined the probability for an individual to stay at the current node $i$ at each timestep consistently as ${p}_{s}=0.5$, and the probability of moving from node $i$ to $j$ as $(1-{p}_{s})\times {w}_{ij}/{\Sigma }_{j}{w}_{ij}$ (where ${w}_{ij}$ is the weight of the link between node $i$ and $j$). We simulated pathogen transmissions in the population using an SIR epidemic model (Keeling and Eames 2005; Kermack and McKendrick 1927), where each individual is either susceptible (S), infectious (I) or recovered (R). We randomly set one individual (1% of the population) as infectious, and at each timestep, simulated infected individual infecting each of its susceptible neighbours in the same patch (if any) with probability $\pi =0.05$. Individuals recovered and acquires immunity with the probability $\rho =0.01$. We tracked the percentages of infected individuals in the simulated populations over time, and compared the mean percentages of infections observed from 100 replications.

We show that the landscape properties play a role in mediating the pathogen transmission dynamics. Importantly, large- (landscape geometry) and local- (the propensity for patches to be more connected) scale characteristics work together to shape the transmission dynamics of a simulated pathogen. A weaker relationship between inter-patch potential connectivity and inter-patch configurational features (as modelled by a lower $\lambda$ value) increases the scale of global disease outbreaks, but this effect is most strongly realized in landscapes with a larger aspect ratio (Fig. 5a), such as a forest habitat in a narrow valley. Our results relate to existing literature linking social (or contact) network structures to patterns of disease transmissions—the stronger tendency for patches to be connected (i.e. $\lambda =0.001$) and squarer landscapes (i.e. $L=5$) typically decrease the path length, or diameter (Fig. 5b), and clustering (Fig. 5c) of the habitat networks, and correspondingly, increase the pathogen outbreak size in simulated populations. Our results complement recent work demonstrating that the fragmentation of animal habitats can impact the transmission dynamics of pathogens (Silk et al. 2019), extending it by showing that the shape as well as the internal potential connectivity of a habitat is important.

## Discussion

We present a multi-dimensional framework for simulating networks that can realistically capture the diverse physical configurations of animal habitats. Our model provides a tool to develop a more mechanistic understanding of the role of habitat configuration in modulating population processes and outcomes. Such modulating effects are likely to be widespread—for example, we have demonstrated that the structure of the habitat network can have consequences on pathogen transmission, in line with predictions from studies of social networks. Developing such mechanistic knowledge is critical as natural animal populations face increasingly rapid changes in their habitats, which have ecological and evolutionary consequences. For instance, habitat change can affect the magnitude of competition (Calizza et al. 2017) and the spread dynamics of pathogens (Bloomfield et al. 2020), information (Betts et al. 2008), or genes (Keller and Largiader 2003). By taking a bottom–up and spatially explicit approach to capture habitat configurations, our model can be tuned to approximate the potential connectivity of specific habitat configurations for a given species. From these, researchers can produce a range of alternative and realistic scenarios to explore the consequences of different features of the habitat on population processes, such as changes in the spatial arrangements of resource patches and physical barriers to movement.

The fundamental role of the physical environment on animal populations makes the evaluation of the consequences of habitat configuration relevant to both theorists and empiricists. If we do not explicitly consider habitat configuration, we risk missing the importance of its contribution to biological processes. For example, simulating social networks of large populations without considering spatial dependencies could produce networks that are more connected than they should be (i.e. without considering the spatial constraints on social interactions). Doing so can misrepresent the biological processes that network structure shapes, such as the transmissions of pathogens (White et al. 2018; Wilkinson et al. 2018), information (Aplin et al. 2015), genes (Vähä et al. 2007). By contrast, current geometric network models, which are spatially-dependent, may largely overestimate the spatial clustering of habitat components because it does not allow for rare long-distance connections or missing connections among close patches by forcing all closely located components to be connected. The need for models tailored to simulate habitat networks has been highlighted by recent studies that modelled specific habitat scenarios. For example, Carraro et al. (2020) proposed a toolkit for generating networks to capture the topological features of real riverine habitats to understand their role in shaping the key processes in freshwater ecology and evolution. The AHN model herein proposed is a more general and flexible framework for depicting habitat potential connectivity. Notably, when simulating networks, the AHN model allows any spatial distributions of habitat components in any landscape (i.e. by tuning $A$ and/or $L$), and provides a cluster of probability curves (i.e. by tuning the $\mu$ and/or $\lambda$ in the ${P(D}_{ij})$) to model the diverse patterns of potential connectivity among habitat components. In addition, the AHN model can generate alternative representations of the potential connectivity of given habitats, and allows control over the deviations of alternative scenarios from the specific habitats that can be used to generate scenarios. Thus, our proposed model can generate realistic habitat scenarios that are biologically meaningful.

There are many useful applications in generating realistic animal habitat scenarios. For example, there is growing interest in understanding the interplay between habitat physical configurations and individuals’ behaviours to predict the persistence of animal populations (Snijders et al. 2017), and explain the structure and composition of ecological communities (Altermatt and Holyoak 2012; Carraro et al. 2020). Rapid habitat changes are also a major threat to wildlife, as they can alter the movement patterns of individuals which may have consequences for populations (Collingham and Huntley 2000; Todd et al. 2009). As habitat changes, individual animals can experience different spatial distributions of resources and risks, which in turn can alter the patterns of both intraspecific (Banks et al. 2007) and interspecific (Farine et al. 2015; Meise et al. 2019) interactions among individuals, as well as other processes such as dispersal patterns and gene flow (Wey et al. 2015). In population ecology, for example, changes in habitat physical configurations can reduce rates of movements among neighbouring subpopulations, which potentially reduces gene flow at the scale of meta-populations (Keller and Largiader 2003) and impacts the persistence of populations (Frankham 2005). Likewise, changes in habitat physical configurations could alter the transmission of information within social networks (Barkoczi and Galesic 2016; Franz and Nunn 2009; Whitehead and Lusseau 2012) and other complex behavioural traits to specific social groups (Nowak et al. 2010; Ohtsuki et al. 2007; Stilwell et al. 2020). Furthermore, altered habitat physical configurations imply potential changes to the transmission dynamics of pathogens across populations (Green et al. 2006; Keeling et al. 2010; Riley 2007; Silk et al. 2019). Our simulations show that, as animal social networks, animal habitat network structures play an important role in shaping pathogen transmission, thus highlight a fundamental link between the physical habitat environments and emergent biological processes, such as the evolutionary dynamics of cooperation (Stilwell et al. 2020) and animal culture (Gruber et al. 2019; Somveille et al. 2018).

Understanding how habitat physical configuration interacts with behavioural and/or demographic dynamics is crucial to assess how vulnerable wild animal populations—and the ecological communities that they are part of (Ryser et al. 2019)—are to the consequences of habitat changes. Our model provides the necessary first step to integrating animal movement at various spatial scales into existing quantitative frameworks. We have demonstrated that the local properties of connectivity and large-scale properties of the landscape can work together to shape population outcomes, such as the spread of pathogens. Such insights can help us to make better predictions or generate new hypotheses on how population or community structures and dynamics are shaped by the physical configurational features of habitats, and how populations or communities might respond to changing physical habitat environments.

## Supplementary Information

Below is the link to the electronic supplementary material.

Supplementary file1 (PDF 252 KB)

## Data Availability

Empirical data used are available from Friesen et al. (2019).

## Code availability

The code for implementing the model is available in the R package *AnimalHabitatNetwork* on the CRAN and GitHub (https://github.com/ecopeng/AnimalHabitatNetwork, where the supplementary file *AnimalHabitatNetwork/Examples/Examples.md* for illustrating the use of our model is also deposited). The code for the simulations is available at https://github.com/ecopeng/Simulation_Code_AHN.
