# Supplementary material for: The role of habitat configuration in shaping animal population processes: a framework to generate quantitative predictions
Source: Oecologia. 2021 Jun 22;196(3):649–65. doi: 10.1007/s00442-021-04967-y (PMC8292241; doi:10.1007/s00442-021-04967-y)
Supplement: Supplementary file 1 — Supplementary file1 (PDF 252 KB) [file 442_2021_4967_MOESM1_ESM.pdf]

APPENDIX

The role of habitat configuration in shaping animal population processes: a framework to generate quantitative predictions

Peng He[1, 2, 3, 4, #], Pierre-Olivier Montiglio[5], Marius Somveille[6, 7], Mauricio Cantor[2, 4, 8, 9], Damien R. Farine[1, 2, 4, +]

[1] Department of Collective Behavior, Max Planck Institute of Animal Behavior, Konstanz, Germany

[2] Centre for the Advanced Study of Collective Behaviour, University of Konstanz, Konstanz, Germany

[3] Department of Biology, University of Konstanz, Konstanz, Germany

[4] Department of Evolutionary Biology and Environmental Science, University of Zurich, Zurich, Switzerland

[5] Department of Biological Sciences, University of Quebec at Montreal, Montreal, QC, Canada

[6] Birdlife International, The David Attenborough Building, Cambridge, UK

[7] Department of Biology, Colorado State University, Fort Collins, CO, 80523 USA

[8] Department for the Ecology of Animal Societies, Max Planck Institute of Animal Behavior, Konstanz, Germany

[9] Departamento de Ecologia e Zoologia, Universidade Federal de Santa Catarina, Florianópolis, Brazil

[#] phe@ab.mpg.de

[+] damien.farine@ieu.uzh.ch

Authors' ORCID

PH: 0000-0002-7176-701X

POM: 0000-0002-1313-9410

MS: 0000-0002-6868-5080

MC: 0000-0002-0019-5106

DRF: 0000-0003-2208-7613

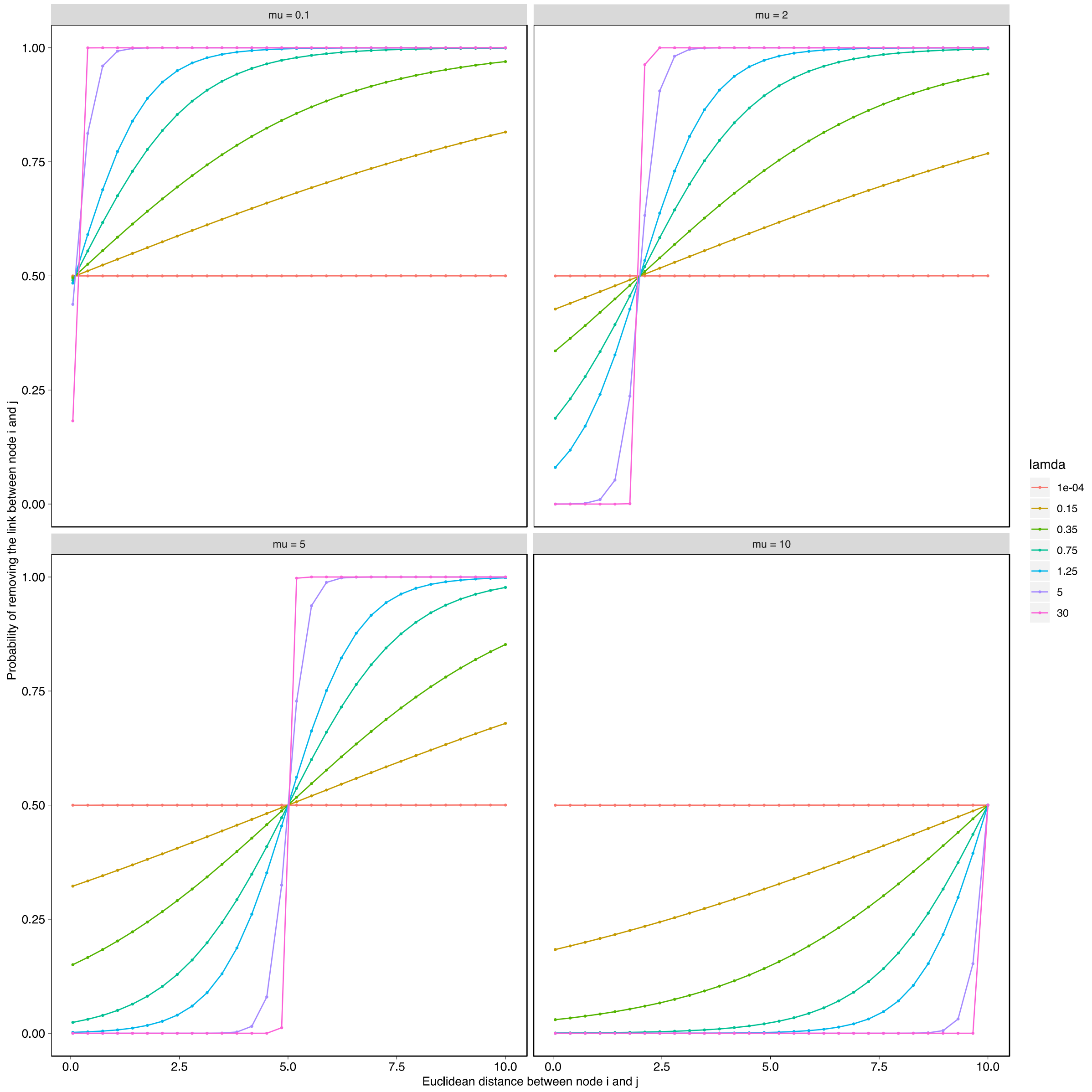

Fig. A1 The effects of the parameter λ (lamda) and μ (mu) on the link filtering out probability function $P(D_{ij}) = [1 + \exp(-\lambda(D_{ij} - \mu))]^{-1}$ in the AHN model. As λ increases, the Euclidian distance between habitat components has an increasing effect on the probability of removing links from the initial complete network, while as μ increases, the probability decreases.

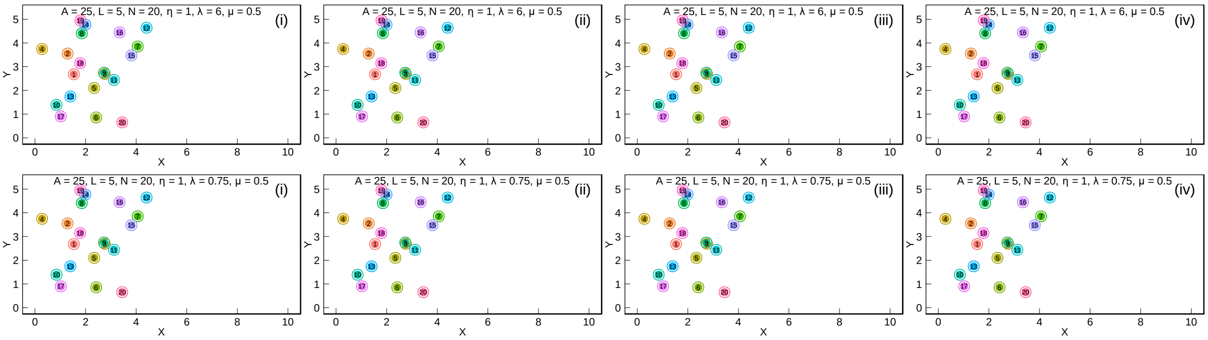

Fig. A2 The effects of the parameter λ in the model on the structures of the resulting animal habitat networks. As λ decreases, the density of the habitat network tends to increase.

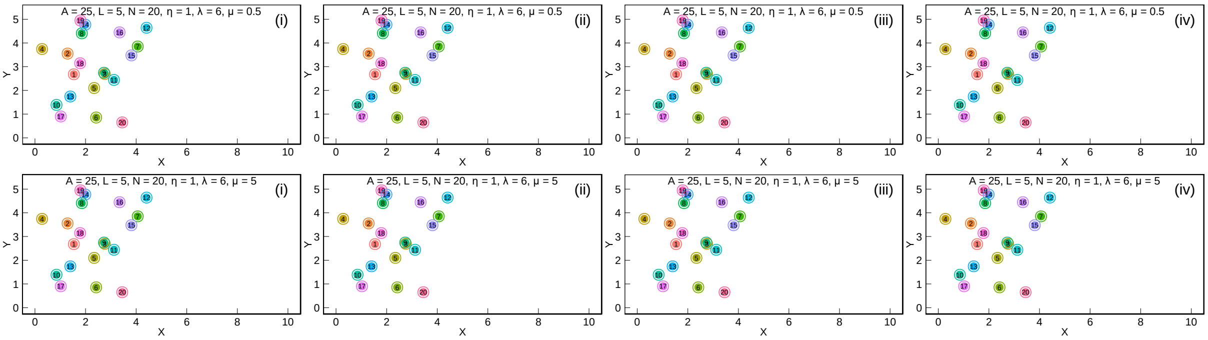

Fig. A3 The effects of the parameter μ in the model on the structures of the resulting animal habitat networks. As μ increases, the habitat network tends to be more densely connected (less links will be removed from the initial complete network).

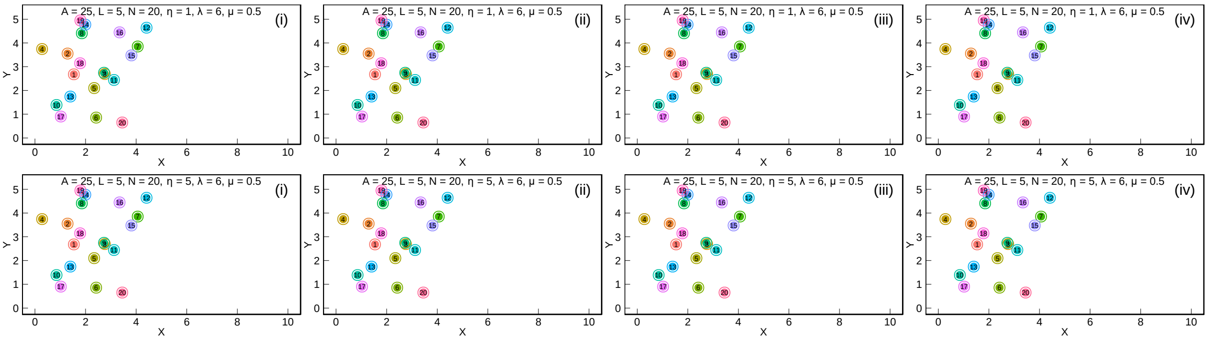

Fig. A4 The effects of the parameter η in the model on the structures of the resulting animal habitat networks. An increasing η has an increasing effect on the weights of rewiring links.

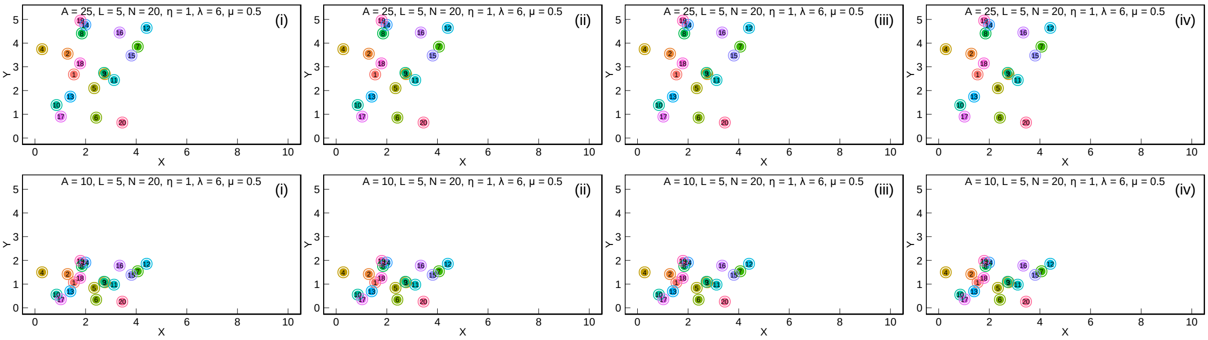

Fig. A5 The effects of the parameter A in the model on the structures of the resulting animal habitat networks. As A decreases, the spatial extent at which the habitat network is defined decreases and the network tend to be more densely connected (due to a decrease in the Euclidean distances between habitat components).

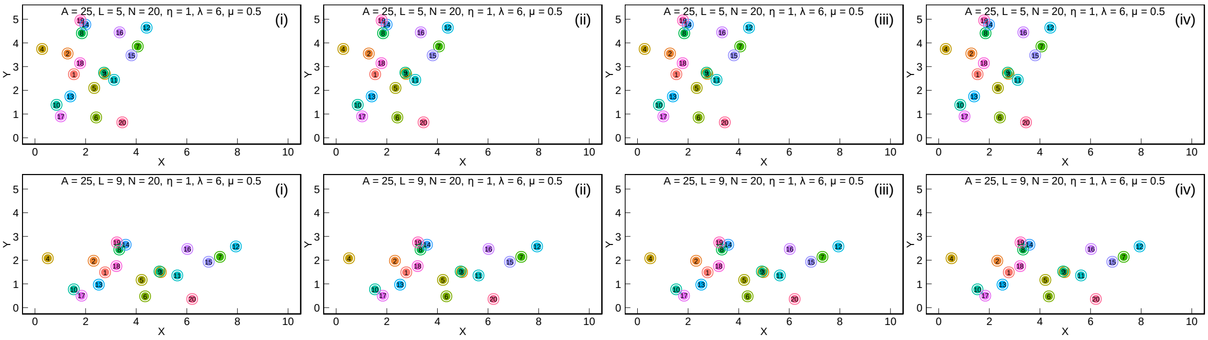

Fig. A6 The effects of the parameter L in the model on the structures of the resulting animal habitat networks. As L increases, the linearity of the habitat network increases.

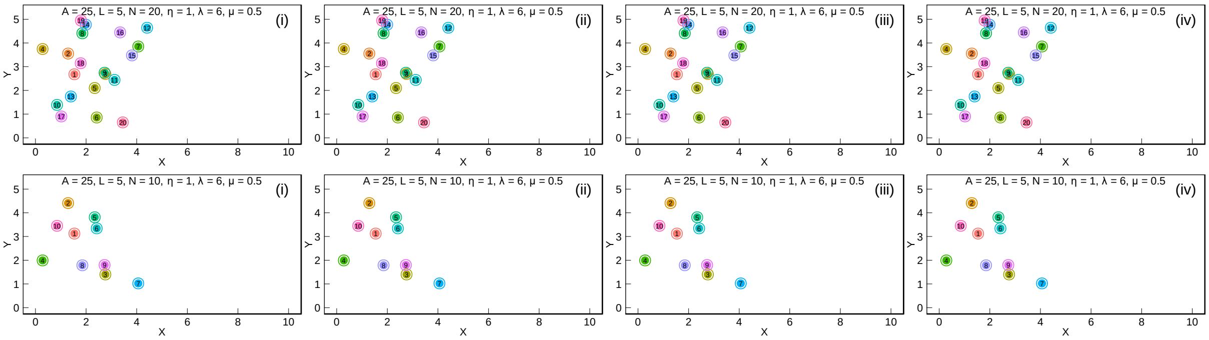

Fig. A7 The effects of the parameter N in the model on the structures of the resulting animal habitat networks. As N decreases, the size and configurational complexity of the network decrease, while the spatial resolution at which the habitat network is defined increases.
